# The expression of inhibitor of bruton’s tyrosine kinase gene is progressively up regulated in the clinical course of chronic lymphocytic leukaemia conferring resistance to apoptosis

**DOI:** 10.1038/s41419-017-0026-3

**Published:** 2018-01-09

**Authors:** Francesco Albano, Federico Chiurazzi, Selena Mimmi, Eleonora Vecchio, Arianna Pastore, Clementina Cimmino, Camilla Frieri, Enrico Iaccino, Antonio Pisano, Gaetanina Golino, Giuseppe Fiume, Massimo Mallardo, Giuseppe Scala, Ileana Quinto

**Affiliations:** 1 Department of Experimental and Clinical Medicine, University “Magna Graecia” of Catanzaro, Catanzaro, Italy; 2 Department of Clinical Medicine, University “Federico II” of Naples, Naples, Italy; 3 Department of Molecular Medicine and Medical Biotechnologies, University “Federico II” of Naples, Naples, Italy

## Abstract

Chronic lymphocytic leukaemia (CLL) is the most common B-cell malignancy with a variable clinical outcome. Biomarkers of CLL progression are required for optimising prognosis and therapy. The Inhibitor of Bruton’s tyrosine kinase—isoform α (*IBTK*α) gene encodes a substrate receptor of Cullin 3-dependent E3 ubiquitin ligase, and promotes cell survival in response to the reticulum stress. Searching for novel markers of CLL progression, we analysed the expression of *IBTK*α in the peripheral blood B-cells of CLL patients, before and after first line therapy causing remission. The expression of *IBTK*α was significantly increased in disease progression, and decreased in remission after chemotherapy. Consistently with a pro-survival action, RNA interference of *IBTK*α increased the spontaneous and Fludarabine-induced apoptosis of MEC-1 CLL cells, and impaired the cell cycle of DeFew B-lymphoma cells by promoting the arrest in G0/G1 phase and apoptosis. Consistently, RNA interference of *IBTK*α up regulated the expression of pro-apoptotic genes, including *TNF*, *CRADD, CASP7, BNIP3 and BIRC3*. Our results indicate that *IBTK*α is a novel marker of CLL progression promoting cell growth and resistance to apoptosis. In this view, *IBTK*α may represent an attractive cancer drug target for counteracting the therapy-resistance of tumour cells.

## Introduction

Chronic lymphocytic leukaemia (CLL) is the most common adult leukaemia in Western countries with an incidence of 4.6 per 100,000 new cases per year (http://www.who.int/selection_medicines/committees/expert/20/applications/CLL.pdf). It is diagnosed by routine blood test showing monoclonal B-lymphocytosis that is typically CD5^+^^1^ and persists for more than 3 months with progressive accumulation of tumour B-cells in bone marrow and lymphoid tissues. CLL patients may present a stably asymptomatic disease without requiring any treatment in their lifetime, or may suffer of rapid disease progression with poor outcome. Sequencing of immunoglobulin heavy chain variable sequence (*IGHV*) of B-cell receptor (BCR) classifies CLL in mutated (M-CLL) and unmutated (U-CLL) subtypes, depending on more or less than 2% mutations compared to germline^1,2^. U-CLL and M-CLL origin from pre-germinal centre (GC) or post-GC CD5^+^ B-cells, respectively, being U-CLL the less common (1%) subtype^3^. *IGHV*-based classification has been used for predicting the clinical course of disease, as U-CLL is associated with poorer outcome compared to M-CLL. However, emerging evidence indicates that prognosis cannot be restricted to the *IGVH* mutation status, and great effort is still required for identifying molecular markers of disease progression. The enhanced expression of *CD38*, *Lipoprotein lipase* (*LPL*) and *Zeta-chain-associated protein kinase 70* (*ZAP70*) was associated to U-CLL with rapid fatal outcome^4–7^. Nevertheless, these genes were not proved to be reliable biomarkers for evaluating the clinical course of CLL and the effectiveness of therapy.

The *Inhibitor of Bruton’s tyrosine kinase* (*IBTK*) gene maps at 6q14.1 chromosomal region^8,9^, proximally to the 6q deletion hotspots of CLL^10–12^. Deletions in the *IBTK* genomic region occur in Richter’s Transformation of CLL to relapsed diffuse large B-cell lymphoma (DLBCL), a very aggressive form of non-Hodgkin lymphoma (NHL)^13^. The *IBTK* gene encodes the proteins isoforms IBtkα, IBtkβ and IBtkγ and the pre-miR-IBTK3^9,14^. The 26 kDa IBtkγ protein was originally characterised as an inhibitor of Bruton’s tyrosine kinase (Btk)^8,15^, an essential enzyme for BCR signalling. The 150 kDa IBtkα protein is the most abundant isoform that is highly expressed in B-lymphoid tissues^9,16^. It acts as substrate receptor of the Cullin 3-dependent E3 ligase, and promotes the K-48 ubiquitination coupled to proteasomal degradation of Pdcd4, a translation inhibitor, resulting in the increased cap-dependent and independent translation^16^. RNA interference of *IBTK*α affected the wide genome expression and RNA splicing in HeLa and K562 cells in cell-type specific manner^17^. Collectively, these findings indicate that IBtkα has pleiotropic effects in protein synthesis/turnover and RNA metabolism.

There is evidence that IBtkα is involved in cell survival and tumour growth. In fact, RNA interference of *IBTK*α reduced the viability of DLD-1 K-Ras-positive colorectal cancer cells^18^. Further, the exposure to the industrial pollutant titanium dioxide increased the production of IBtkα in human bronchial epithelial cells^19^. Thapsigargin and tunicamycin, two inducers of endoplasmic reticulum stress, also increased the expression of IBtkα in HeLa cells, and this event was required for cell survival^20^. Being highly expressed in B-lymphoid tissues, IBtkα could sustain B-cell survival, and eventually be involved in B-tumorigenesis when up regulated. Consistently with this hypothesis, differential methylation of the *IBTK* genomic region was reported for U-CLL and M-CLL, suggesting a possible correlation between the *IBTK*α expression level and aggressiveness of disease^21^.

## Results

### The expression of IBTKα is up regulated in CLL progression

**Fig. 1:**
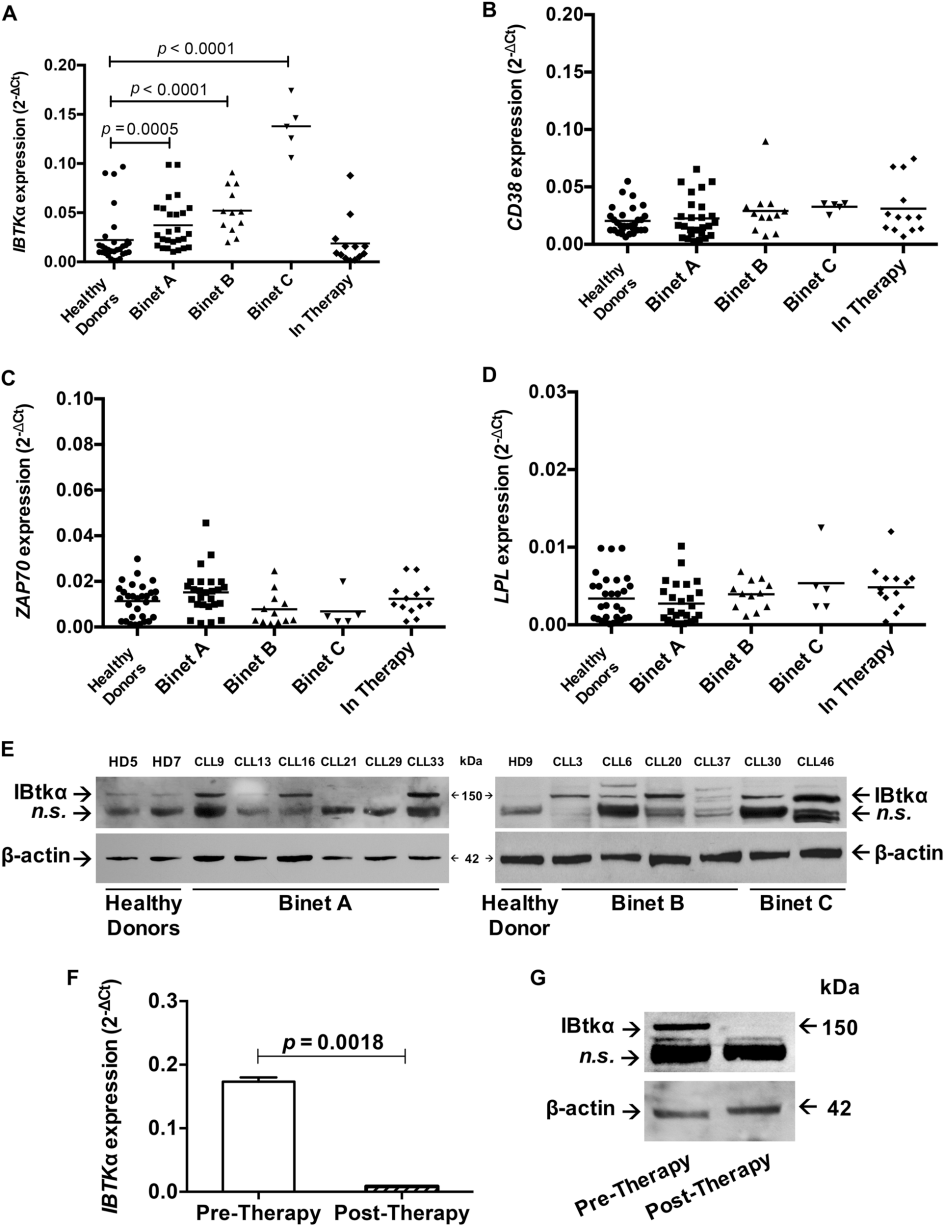
Expression of IBTKα, LPL, CD38 and ZAP70 in CLL patients . **a**–**d** B-cells were isolated from healthy donors (HD), and CLL patients of Binet A, B, C and In-Therapy groups. Total RNA was analysed by RT-qPCR for the expression of *IBTK*α **a**, *CD38* **b**, *ZAP70* **c**, and *LPL* **d**. β-*ACTIN* expression was measured for normalisation. Statistical analysis was performed using Mann–Whitney test (GraphPad Prism 6). **e** Protein extracts from healthy donors (HD) and CLL patients of Binet A (CLL9, 13, 16, 21, 29, 33), Binet B (CLL3, 6, 20, 37) and Binet C (CLL30, 46) stages. Proteins were separated by 4-12% NuPAGE Novex Gels (ThermoFisher Scientific) and analysed by Western blotting with antibodies against IBtkα (ThermoFisher Scientific #PA5-24224) and β-actin (Cell Signalling #3700S). **f** *IBTK*α gene expression in CLL11 patient before and after first line therapy with Rituximab and Bendamustine. Total RNA was analysed by RT-qPCR. Mean values (*n* = 3) ± SE are shown. Statistical analysis was performed using Unpaired *t*-test with Welch’s correction (GraphPad Prism 6). **g** IBtkα protein content in CLL11 patient before and after first line therapy with Rituximab and Bendamustine. Protein extracts were analysed by Western blotting with antibodies against IBtkα (ThermoFisher Scientific #PA5-24224) and β-actin (Cell Signalling #3700 S). n.s. indicates non-specific bands

This study was aimed to analyse the expression of *IBTK*α in B-lymphocytes of CLL patients at different stages of disease, before and after first line therapy. Total RNA of CD19^+^ B-cells was analysed by Real-time PCR for the expression of *IBTK*α and some markers of CLL aggressiveness, including *LPL*, *CD38* or *ZAP70* genes (Supplementary Table 2). The expression of *IBTK*α gene progressively increased in Binet A (*p* = 0.0005), Binet B (*p* < 0.0001) and Binet C (*p* < 0.0001) groups compared to healthy donors, with the highest expression in Binet C group (Fig. 1a). No difference of *IBTK*α expression was observed between healthy donors and patients after therapy causing remission of disease (Fig. 1a). In the same samples, no significant differences in expression levels of *LPL*, *CD38* and *ZAP70* were observed between healthy donors and CLL groups (Figs. 1b–d). These genes were previously reported to be up regulated in the U-CLL subtype with the poorest outcome^4–7^. Based on *IGHV* sequencing^22^, ten CLL patients with the highest *IBTK*α expression (CLL 2, 3, 4, 5, 11, 14, 22, 25, 26, 31) belonged to the M-CLL subtype, which might explain the lack of up-regulation of *LPL*, *CD38* and *ZAP70* genes in our CLL samples. In the group of patients in therapy with remission, the expression of *IBTK*α dropped to the healthy control level (Fig. 1a), indicating that the hyper-expression of *IBTK*α was associated with the presence of tumour cells.

IBtkα is a substrate receptor of Cul3-dependent E3 ubiquitin ligase and undergoes K48-ubiquitination coupled to proteasomal degradation^16^. Thus, we evaluated whether the IBtkα protein level correlated with the increased *IBTK*α RNA expression in CLL samples. To this end, B-cell extracts of twelve randomly chosen CLL patients were analysed by Western blotting for the content of IBtkα protein. Compared to healthy donors, the IBtkα protein significantly increased in 3 out of 6 Binet A patients, 5 out of 5 Binet B patients, and 2 out of 2 Binet C patients (Fig. 1e), indicating a parallel increase in both *IBTK*α RNA and protein levels in most of CLL patients (compare Fig. 1e and Supplementary Figure 2). The limiting amount of CLL cells did not allow extending the Western blotting analysis of IBtkα protein to other patients. To overcome this problem, we developed an intracellular flow cytometry protocol for staining IBtkα with a specific antibody. We confirmed that the content of IBtkα protein progressively increased in three CLL patients of Binet stage A (CLL13), B (CLL20) and C (CLL46) with the aggressiveness of disease (Supplementary Figure 3).

In one patient (CLL11 of Binet B group) undergoing first line therapy, we could analyse both *IBTK*α gene and protein expression before and after treatment with Rituximab and Bendamustine. A strong reduction of *IBTK*α transcripts (about 100-folds, *p* = 0.0018) and IBtkα protein was observed after therapy causing remission (Figs. 1f, g). Even though this analysis was restricted to one single patient, it provides additional evidence that IBtkα is a tumour-associated marker and could be a measure of effectiveness of therapy.

### Lack of IBTKα sensitises CLL cells to spontaneous and fludarabine-induced apoptosis

**Fig. 2:**
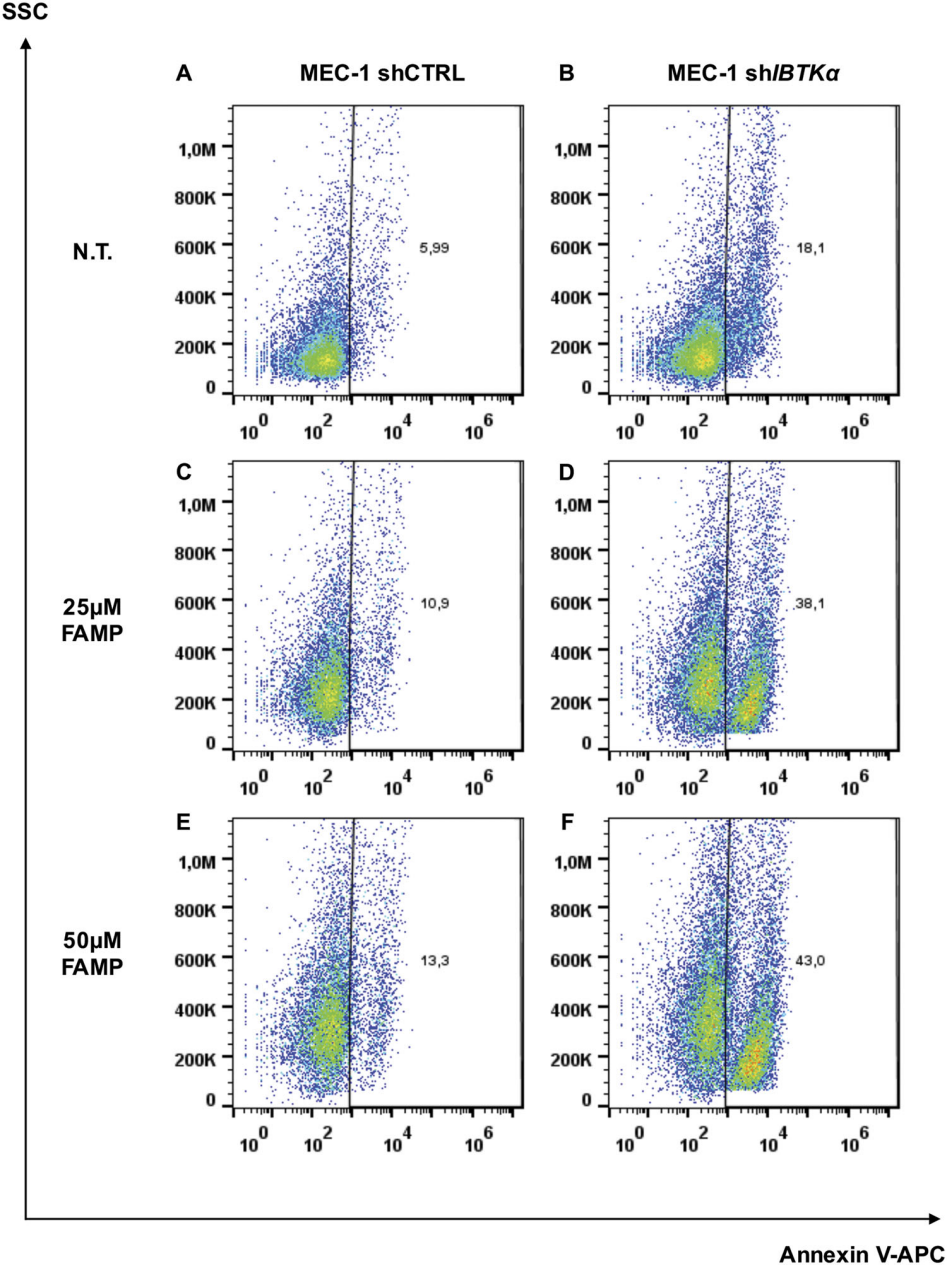
IBTKα RNA interference increases spontaneous and fludarabine–induced apoptosis in MEC-1 CLL cells MEC-1 cells were transduced with TurboGFP-shCTRL or TurboGFP-sh*IBTK*α (Sigma-Aldrich), and after 5 days were left untreated (N.T.) **a**, **b** or 72 h-treated with Fludarabine at 25 µM **c** and **d** or 50 µM **e**, **f**. Then, cells were collected, stained with Annexin-V-APC and analysed by flow cytometry. Binding of Annexin-V-APC was measured for GFP-positive cells, as positive control of transduction. A representative experiment of three independent experiments is shown. Statistically significant differences were assessed using Mann–Whitney test (GraphPad Prism 6), as follows: **a** vs. **b** *p* = 0.0043; **c** vs. **d** *p* = 0.0002; **e** vs. **f** *p* = 0.0001

The purine analogue Fludarabine (FAMP) is among the most effective chemotherapeutic agents for first-line and second-line treatment of CLL^23^. FAMP-resistance occasionally occurred in tumour cells^24^. To address the question whether IBtkα was involved in mechanisms of FAMP-resistance, we analysed the effect of *IBTK*α RNA interference on FAMP-sensitivity of MEC-1, a CLL-derived cell line peculiarly resistant to apoptosis induced by chemotherapeutic agents^25^. We first transduced MEC-1 cells with lentiviral particles expressing sh*IBTK*α or shCTRL together with Green Fluorescent Protein (GFP), for monitoring cell transduction efficiency. Forty eight-hours post-transduction, MEC-1 cells were left untreated or treated with 25 or 50 µM FAMP for 72 h, within a dose range lower than 100 µM inducing apoptosis^26^. After treatment, GFP-positive cells were analysed by FACS for the binding of Annexin V-APC as measure of apoptosis. RNA interference by sh*IBTK*α caused 3-folds increase in apoptosis compared to shCTRL (18.1% apoptosis in sh*IBTK*α compared to 5.99% in shCTRL; *p* = 0.0043) (Figs. 2a, b). A similar increase in apoptosis was observed in absence of *IBTK*α when cells were treated with lower FAMP concentration (25 µM) (38.1% apoptosis in sh*IBTK*α compared to 10.9% in shCTRL) (Fig. 2c, d; *p* = 0.0002), and higher FAMP concentration (50 µM) (43% apoptosis in sh*IBTK*α compared to 13.3% in shCTRL (Fig. 2e, f; *p* = 0.0001). These results collectively indicated that the lack of IBtkα sensitised MEC-1 cells to apoptosis, with or without FAMP treatment, suggesting that IBtkα inhibition could improve the efficacy of chemotherapy.

### IBTKα RNA interference impairs the cell growth by increasing G0/G1 and apoptotic cells of cell cycle and up regulating the expression of pro-apoptotic genes

**Fig. 3:**
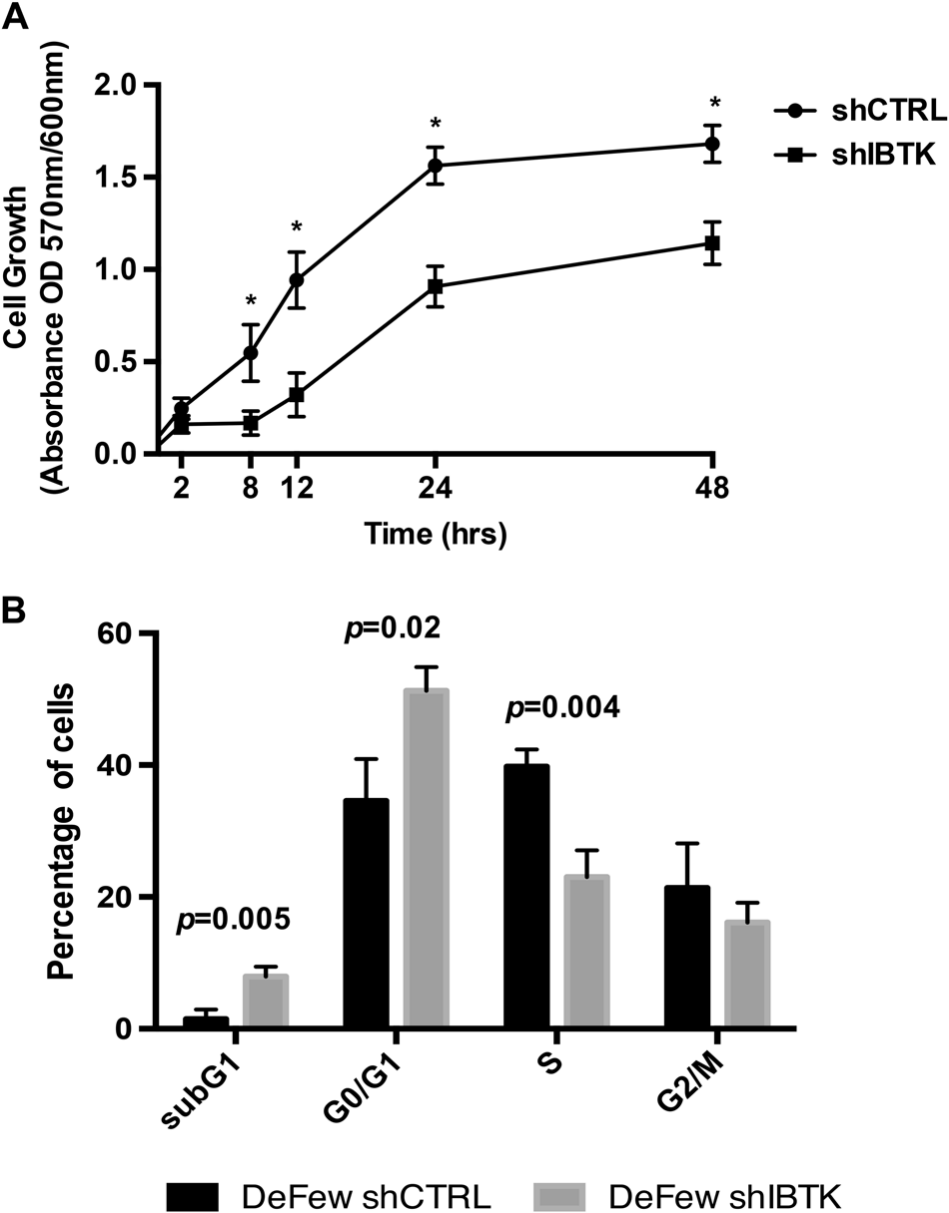
IBTKα RNA interference impairs the B-cell growth **a** The growth of DeFew cells, with or without *IBTK*α RNA interference, was measured by resazurin-based colorimetric assay. Mean values (*n* = 5) ± SE are shown. Doubling time was about 40 h for sh*IBTK*α-transduced cells compared to 24 h for shCTRL-transduced cells. Statistical significance was determined using the Holm-Sidak method, with alpha = 5.000%. Each row was analysed individually, without assuming a consistent SD (GraphPad Prism 6). The asterisk indicates statistical significance as follows: at 8 h *p* = 0,0167; at 12 h *p* = 0.005; at 24 h *p* = 0.0016; at 48 h *p* = 0.0037. **b** Cell cycle of DeFew, with or without *IBTK*α RNA interference, was analysed by Propidium Iodide staining. Mean values (*n* = 3) ± SE are shown. Statistical analysis was performed using Unpaired *t*-test with Welch’s correction (GraphPad Prism 6)

Previous reports indicated that IBtkα was involved in the cellular stress response^18–20^. To get further insights into the pro-survival role of IBtkα, the *IBTK*α gene was silenced in DeFew cells by RNA interference with lentiviral transduction of sh*IBTKα* or shCTRL (Supplementary Figure 4), and the cell growth was analysed by resazurin-based colorimetric assay. Cells silenced for *IBTK*α had a doubled replication time compared to unsilenced cells (Fig. 3a). To understand whether the slower growth of *IBTK*α-silenced cells was due to delay in cell replication or increase in cell death, we analysed the cell cycle using Propidium Iodide staining. As compared to shCTRL, the number of sh*IBTK*α-transduced DeFew cells was significantly reduced in S-phase (from 40 to 20%; *p* = 0.004), and increased in G0/G1 population (from 38 to 57%; *p* = 0.02) and subG1 population (from 2 to 10%; *p* = 0.005) (Fig. 3b). These data indicate that lack of *IBTK*α caused a partial block of the cell cycle in G0/G1 phase together with the increase of apoptotic cells.

**Fig. 4:**
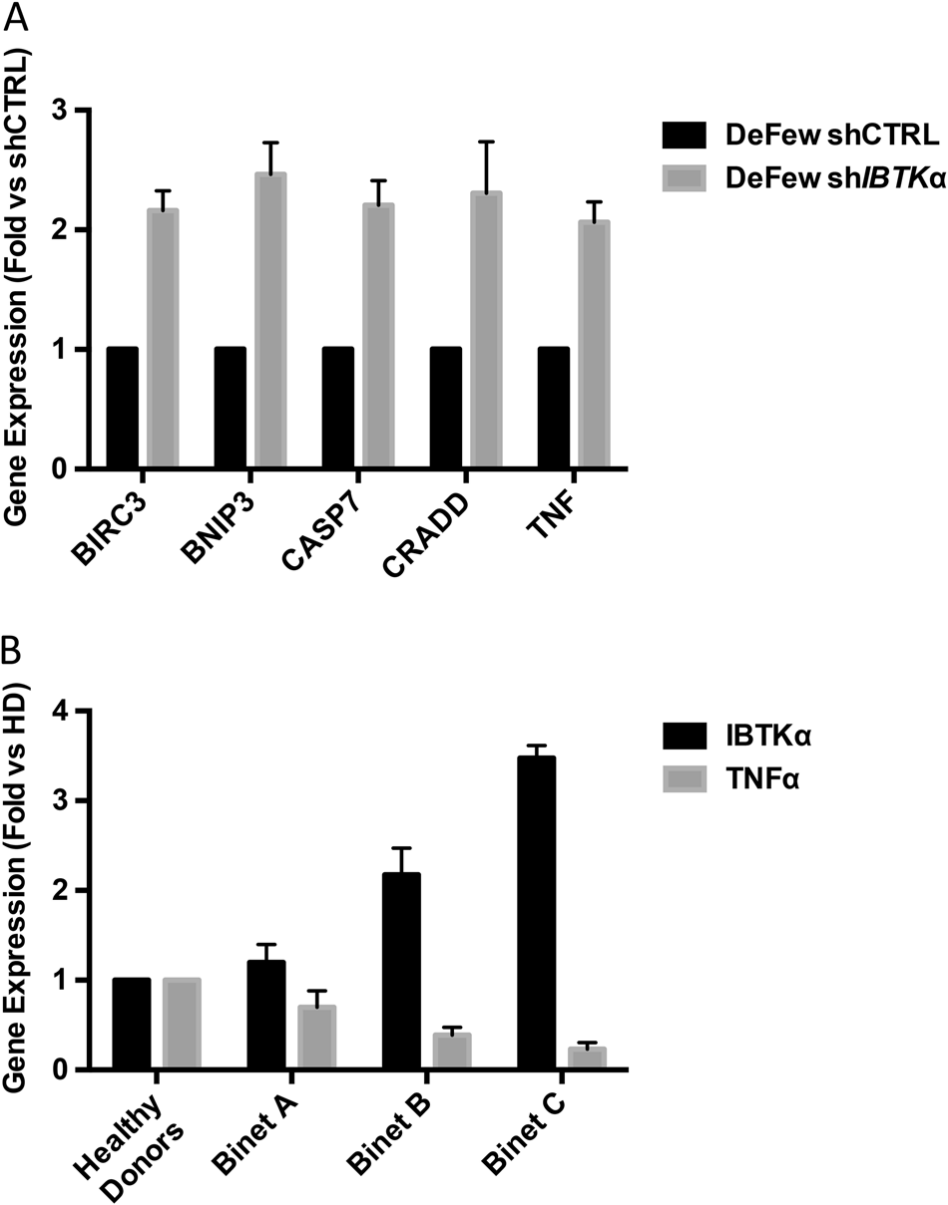
IBTKα expression inversely correlates with TNFα expression in the CLL progression **a** DeFew cells were transduced with shCTRL or sh*IBTK*α, and after 5 days the total RNA was analysed by RT-qPCR, using RT^2^ Profiler PCR Array-Human Apoptosis (Qiagen) Values (mean ± SE, *n* = 3) are shown. Mean of *ACTB*, *B2M* and *GAPDH* expression was used for normalisation. **b** B-cells were isolated from healthy donors (HD), and representative patients of Binet A (CLL47), B (CLL43) and Binet C (CLL52) stages. Total RNA was analysed for the expression of *IBTK*α and *TNF*α by RT-qPCR. β-*ACTIN* expression was measured for normalisation. Statistical analysis was performed using Mann-Whitney test (GraphPad Prism 6). Mean values (*n* = 3) ± SE are shown

To address the molecular mechanisms of IBtkα-dependent cell survival, we analysed DeFew cells for the expression of 84 genes involved in apoptosis (Supplementary Table 3), with and without *IBTK*α RNA interference. TNFα and four pro-apoptotic genes of TNFα and NFκB signalling (*CRADD, CASP7, BNIP3 and BIRC3*) were significantly up regulated in absence of IBtkα (Fig. 4a). These results suggested that IBtkα likely inhibited the expression of pro-apoptotic genes, including *TNF*α. To test this hypothesis, we measured the expression of *IBTK*α and *TNF*α genes by Real-Time PCR of total RNA extracted from CLL cells of representative patients of Binet A, B and C stages. A progressive decrease of *TNF*α expression was observed in the clinical course of disease, concomitantly with the increased expression of *IBTK*α (Fig. 4b). This observation was consistent with *TNF*α up regulation in *IBTK*α-silenced DeFew cells (Fig. 4a). Altogether these results suggest that *IBTK*α could promote tumour survival by inhibiting the expression of pro-apoptotic genes.

## Discussion

This study provides the first evidence of IBtkα as prognostic marker of CLL progression. Our conclusions are based on the analysis of *IBTK*α expression in three independent groups of CLL patients that were stratified according to the Binet staging system. One additional group included CLL patients with disease remission after first line therapy. The main objectives were: (a) to determine whether the *IBTK*α expression was modulated in the clinical course of disease and after first therapy; (b) to compare *IBTK*α to prognostic markers of CLL, including *CD38*, *LPL* and *ZAP70* genes; (c) to unveil the potential role of *IBTK*α in CLL progression.

We observed that the *IBTK*α expression was increased in CLL cells concomitantly with the progression of the disease. Conversely, the expression of *IBTK*α diminished in patients after first therapy, jumping to the levels of healthy donors. In the same CLL samples, the expression of *CD38*, *LPL* and *ZAP70* genes was similar to healthy donors. In previous reports the enhanced expression of *CD38*, *LPL* and *ZAP70* was reported in U-CLL with the poorest outcome^4–7^. In our study, we randomly enroled CLL patients independently of the *IGHV* mutational status. However, according to *IGHV* genetic analysis, CLL patients with the highest *IBTK*α expression and advanced stage of disease were M-CLL, which could explain the differences between our data and previous reports. We also investigated the *IBTK*α expression in the same patient before and after first line therapy with Rituximab and Bendamustine. This patient was M-CLL subtype enroled at a very aggressive stage of disease. First line therapy caused the remission of disease, as monitored by clinical analysis and cytofluorimetry. In this patient, *IBTK*α was hyper-expressed in peripheral blood B cells before therapy, while it was down regulated after therapy. Based on this evidence, *IBTK*α was a sensitive marker of CLL progression and response to therapy.

CLL pathogenesis initiates with the proliferation of precursor B-cells in bone marrow followed by their expansion, clonal selection and transformation in lymph nodes. The correlation between *IBTK*α expression and tumour aggressiveness suggests a pro-tumorigenic function of the IBtkα protein. It is not clear whether the deregulated expression of *IBTK*α contributes to CLL pathogenesis or it is a consequence of altered pathways in B-tumorigenesis. Previous reports indicated that *IBTK*α RNA interference caused the loss of viability and apoptosis of K-Ras-positive colorectal cancer cells^18^ and HeLa cells exposed to endoplasmic reticulum stress^20^, supporting a pro-survival action of IBtkα. Based on the hypothesis that IBtkα could be required for CLL growth and survival, we analysed B-cells viability in presence or absence of *IBTK*α using RNA interference. In DeFew cells, we showed that *IBTK*α silencing delayed the growth kinetic, by arresting the cells in G0/G1 phase of cell cycle and increasing the number of apoptotic cells. We wondered whether RNA interference of *IBTK*α could sensitise B-cells to drug-induced apoptosis. Indeed, the lack of IBtkα enhanced both the spontaneous and Fludarabine-induced apoptosis of MEC-1, a CLL-derived cell line peculiarly resistant to apoptosis^25,26^.

We previously reported that *IBTK*α RNA interference affected the whole genome expression and RNA splicing of several genes in K562 and HeLa cells^17^. Thus, we addressed the question whether IBtkα could regulate the expression of death-related genes in the B-cell context. To this end, we analysed the expression of 84 genes involved in apoptosis, in presence and absence of IBtkα. In DeFew cells, RNA interference of *IBTK*α caused the up-regulation of *TNF, CRADD*, *CASP7*, *BNIP3* and *BIRC3*. The comparative analysis of representative number of CLL patients of Binet A, B and C stages and healthy donors revealed that the increased expression of *IBTK*α correlated with the decreased expression of TNFα in CLL progression, which was consistent with the enhanced expression of TNFα in DeFew cells upon *IBTK*α RNA interference.

The five genes deregulated by IBtkα at the transcriptional levels are linked to the TNFα and NF-κB signalling, which is relevant for B-cell survival and proliferation. *CRADD* and *CASP7* act in TNFα signalling leading to apoptosis through the activation of a cell death signal transduction complex. In particular, CRADD is a protein containing a death domain recruiting Caspase 2 to membrane receptors for death signalling, such as the tumour necrosis factor receptor 1^27^. CASP7 is an inducible caspase by the TNFα receptosomes^28,29^, whose activity was increased in HeLa cells by *IBTK*α RNA interference^20^. Since TNFα, CRADD and CASP7 share the same pro-apoptotic pathway and are commonly up regulated in absence of *IBTK*α, it is feasible that, conversely, the enhanced expression of *IBTK*α in CLL cells could down regulate the expression of these pro-apoptotic genes, thus counteracting apoptosis. Accordingly with this hypothesis, we found that the down-regulation of TNFα expression correlated with the up regulation of *IBTK*α expression in the clinical course of disease. BNIP3 and BIRC3, the other two up-regulated genes by *IBTK*α RNA interference, are tumour suppressors. In particular, BNIP3 is a prognostic marker of breast cancer, whose deficiency was associated with progression to metastasis in certain cancer sub-types^30^. *BIRC3* is a repressor of the non-canonical NF-κB signalling, whose inactivation by mutations occurred in a significant number of FAMP-refractory CLL^31,32^. Again, the increased expression of BNIP3 and BIRC3 by *IBTK*α RNA interference in DeFew cells suggests that *IBTK*α, when hyper-expressed in CLL, could down-regulate the expression of these tumour suppressor genes. Altogether, our results support a pro-survival action of IBtkα through the transcriptional inhibition of pro-apoptotic genes. At this time, it is unclear how IBtkα exactly contributes to the deregulation of apoptotic genes, and further efforts are needed to undercover molecular partners of IBtkα in cancer cells. In this regard, we have previously shown that the IBtkα protein is substrate receptor of Cullin 3-dependent E3 ligase complex and promotes the ubiquitination coupled to proteasomal degradation of the translation repressor Pdcd4^16^. The complexity of IBtkα interactome requires further investigation to characterise other putative targets of IBtkα for degradation^16^. It is reasonable to hypothesise that IBtkα could affect both transcriptome and proteome at least by affecting the stability of transcriptional and translational activator and repressors.

The strict correlation between the *IBTK*α expression and CLL progression indicates that the transcriptional control of *IBTK*α is progressively switched on in the clinical course of disease by not yet identified molecular events. Such epigenetic event could be a part of mechanisms to enrich tumour cells with functions giving a survival advantage. Searching for transcriptional factors activating the *IBTK*α gene in CLL could be relevant to determine the signalling pathways that promote the disease progression. In this context, the development of inhibitors of IBtkα could increase the efficacy of the chemotherapeutic treatment in refractory patients.

## Materials and methods

### Peripheral blood samples

Peripheral blood samples of CLL patients (*n* = 56) were randomly collected at the Haematology Unit of the Department of Clinical Medicine, University “Federico II” of Naples. Peripheral blood samples of healthy donors (HD, *n* = 30) were collected at the Transfusional Centre, University “Federico II” of Naples. All donors gave informed consensus before the sampling. CLL patients were diagnosed by immunophenotyping of peripheral B-lymphocytes (CD5^+^, CD19^+^, CD23^+^) and clinical examinations, and classified according to the Binet staging system^33^. Molecular and clinical data of CLL are summarised in Supplementary Table 1. CLL were grouped as follows: Binet A (*n* = 26); Binet B (*n* = 12); Binet C (*n* = 5). An additional group included in-therapy patients (*n* = 13) of the Binet A, B or C stages, which underwent first line therapy, and showed remission of disease at the time of blood collection based on clinical and haematological parameters, independently of the therapeutic protocols.

CD19^+^ B-cells were isolated by negative selection from whole blood using RosetteSep Human B Cell Enrichment Cocktail (Stem Cell Technology), as previously described^22^, and represented 95% cell population as measured by flow cytometry (Supplementary Figure 1). The *IGHV* regions of ten CLL samples with the highest expression of *IBTK*α were sequenced to determine the mutation level compared to germline, as previously described^22^; these samples carried more than 2% mutations within the *IGHV* region, and thus were defined M-CLL.

### Cells, plasmids, and RNA interference

HEK293T cells were purchased from American Type Culture Collection (Manassas, VA, U.S.A.) and grown in DMEM (Gibco, Invitrogen, Carlsbad, CA) supplemented with 10% heat-inactivated FBS, 2 mM L-glutamine, 100 U/ml penicillin, and 100 μg/ml streptomycin (Gibco). HEK293T cells were used as packaging cells for the production of lentiviral particles containing shRNA. DeFew cells, an EBV-negative B-lymphoma cell line^34,35^, were grown in RPMI 1640 (Gibco) supplemented with 10% heat-inactivated FBS, 2 mM L-glutamine, 100 U/ml penicillin, and 100 μg/ml streptomycin (Gibco). MEC-1 cells, a B-CLL cell line^25^, were purchased from DSMZ (Leibniz, Germany) and were grown in IMDM (Gibco) supplemented with 10% heat-inactivated FBS, 2 mM L-glutamine, 100 U/ml penicillin, and 100 µg/ml streptomycin (Gibco). The plasmids pCMV-dR8.91 and pCMV-VSVG were purchased from AddGene (OneKendall, Cambridge, MA, USA). The lentiviral constructs expressing the *IBTK*α-shRNA (TRCN0000082575) or control shRNA (SHC002) were purchased from MISSION (Sigma Aldrich, St. Louis, MO, USA). The TurboGFP™ version of the *IBTK*α-shRNA and control shRNA vectors carries the lentiviral backbone of vector pLKO.1-puro expressing the TurboGFP gene under the CMV promoter to monitor the transduction efficiency by optical microscopy. The *IBTK*α-shRNA targets the nucleotide sequence from +1534 to +1552 of the *IBTK*α transcript (Ensemble Reference Sequence: ENST00000306270). Lentiviral particles expressing *IBTK*α shRNA or control shRNA were produced by transfection of HEK293T cells^17^. Briefly, HEK293T cells (2 × 10^6^) were transfected with pCMV-dR8.91 (3,5 μg) and pCMV-VSVG (0,7 μg) together with *IBTK*α-shRNA (7 μg), or control-shRNA (7 μg); 48-h post-transfection, cell supernatant was collected. Enzyme-linked immunosorbent assay (ELISA) using anti-p24 antibody measured virions concentration. DeFew or MEC-1 cells (1 × 10^6^) were infected with viral stocks (500 ng of p24) by spinoculation, as previously described^17^. To select cell clones stably expressing *IBTK*α-shRNA or control-shRNA, puromycin (2 μg/mL) was added to cell cultures 48 h after infection, when indicated.

### Real-time PCR

Total RNA was extracted from cells using TRIzol reagent (Thermo Fisher Scientific) according to manufacturer’s protocol. RNA quality was checked by electrophoresis of 1 µg of samples on MOPS-agarose gel. Aliquots (3 µg) of total RNA were subjected to DNase I digestion (Thermo Fisher Scientific*)* and reverse transcribed using SSIV Reverse Transcriptase (Thermo Fisher Scientific) following manufacturer’s instructions. Real-Time PCR was performed with the PowerUP Sybr green master mix (Thermo Fisher Scientific) using a Quant Studio 7 Flex instrument and Fast gene-expression method: 95 °C, 20”; (95 °C, 1”; 60 °C, 20”) × 40 cycles; 95 °C, 15”; 60 °C 1’; 0.05 °C/s up to 95 °C. Real-Time data were analysed using Quant Studio Real-Time PCR Software (Thermo Fisher Scientific). Reactions were carried out in triplicate, and gene expression levels were calculated relatively to * β-Actin* mRNA levels as endogenous control. Real-Time PCR amplification values were reported as 2^−ΔCt^, were ΔCt is Ct^gene under investigation^−Ct^endogenous control^.

For expression analysis of genes involved in apoptosis, total RNA from DeFew cells, silenced or not for *IBTK*α, was analysed by Real-time PCR using RT^2^ Profiler PCR Array-Human Apoptosis (Qiagen). Difference in gene expression was considered significant for more 2-folds up-regulation or down-regulation[38]. We did not consider genes with Ct values ranging from 30 to 40 in shCTRL cells. The primers used for *IBTK*α, *LPL*, *CD38*, *ZAP70*, and *TNF*α genes are listed in Supplementary Table 2.

### Protein extracts and western blotting

CLL samples were processed for Western blotting analysis as previously described^36^. Briefly, cells were lysed in RIPA buffer, containing 1% NP-40, 10 mM Tris-HCl, 150 mM NaCl, 0,5% Na-deoxycholate and 1 mM EDTA, supplemented with the protease inhibitor cocktail (complete-mini EDTA-free tablets—Roche) on ice for 30 min. Lysates were clarified by 30 min-centrifugation at 14,000x*g*, at 4 °C. Western blotting was performed by suspending protein aliquots in loading buffer (Thermo Fisher Scientific), resolved on Novex^®^ NuPAGE^®^ SDS-PAGE Precast Bis-Tris Gels 4 to 12%, transferred to PolyVinylidene-DiFluoride membrane (Millipore, Bedford, MA, USA), and incubated with primary antibodies followed by incubation with horseradish-peroxidase-linked mouse or rabbit IgG (1:2000) (GE Healthcare Amersham, Little Chalfont, UK) in PBS containing 5% non-fat dry milk (Bio-Rad Laboratories). Proteins were detected by chemiluminescence using the Immobilon Western Chemiluminescent HRP substrate (Millipore). Primary antibodies were purchased from Thermo Fisher Scientific (anti-IBtkα, PA5-24224) and Cell Signalling (anti- β-Actin, #3700S).

### Cell proliferation assay

DeFew cells, stably silenced or not for *IBTK*α by RNA interference, were suspended in RPMI 1640 and 5 replicates were plated in 96-wells microtiter plates at the concentration of 1 × 10^4^ cells/100 µl/well. Ten µl of Presto-blue cell viability reagent (Thermo Fisher Scientific) was added to each well at 10% final concentration. PrestoBlue reagent is soluble, non-toxic and quickly reduced by metabolically active cells, providing a quantitative measure of viability and cytotoxicity. Absorbance relative to reagent reduction by cells was measured by optical density at different time points in order to obtain growth kinetics of the cells. Optical absorbance was measured at 570 and 600 nm, and reads were normalised to 600 nm. The read at time 0 (T0) was performed after 10 min of incubation and cell proliferation was monitored for 48 h.

### Propidium iodide assay

Propidium iodide (PI) incorporation was performed as previously described^37^. Briefly, sh*IBTK*α-transduced and shCTRL-transduced DeFew cells (3 × 10^5^) were centrifuged, and pellets were suspended in fluorochrome solution (0.1% sodium citrate, 0.1% Triton X-100, 50 mg/l PI, in deionized water). After 60 min-incubation at 4 °C, cells were analysed using Accury C6 Flow cytometer (Becton Dickinson).

### Flow cytometry

Intracellular IBtkα protein was detected by flow cytometry upon cell staining with anti-IBtkα antibody (Novus NBP1-88512). Briefly, cells were washed with PBS, fixed with 4% Paraformaldehyde, permeabilized with permeabilization buffer (Becton Dickinson Biosciences), washed with PBS and incubated with primary antibody for 30 min at 4 °C. After washing, cells were incubated with anti-rabbit-APC antibody (SouthernBiotech *4050-11S*) for 30 min at 4 °C, and analysed using Accury C6 Flow cytometer (Becton Dickinson).

### Annexin-V assay

Annexin-V assay was performed as previously described^37^. Briefly, sh*IBTK*α-transduced and shCTRL-transduced MEC-1 cells were treated with 25 or 50 µM Fludarabine, or left untreated, for 72 h. At the end of treatments, cells were harvested, washed 2 times with 1x PBS, and suspended by gently vortexing in 100 µl of Annexin-V binding buffer (BD Pharmingen) containing 5 µl of Annexin-V-APC (Becton Dickinson Biosciences), for 15 min at room temperature. After incubation, 400 µl of binding buffer was added and samples were analysed using Accury C6 Flow cytometer (Becton Dickinson). The binding of Annexin-V-APC was evaluated only for GFP-positive cells, as control of shRNA transduction.

### Statistical analysis

Statistical analysis was performed by Mann–Whitney test applying 95% confidence intervals and using a two-tailed *p*-value, with GraphPad Prism 6 software. Differences were considered significant with *p*-value less than 0.05.

## Electronic supplementary material

Supplementary Informations

Supplementary Table 1

Supplementary Table 2

Supplementary Table 3

Supplementary Figure 1

Supplementary Figure 2

Supplementary Figure 3

Supplementary Figure 4

## References

[1] Chiorazzi N, Ferrarini M (2003). B cell chronic lymphocytic leukemia: lessons learned from studies of the B cell antigen receptor. Annu. Rev. Immunol..

[2] Schroeder HW, Dighiero G (1994). The pathogenesis of chronic lymphocytic leukemia: analysis of the antibody repertoire. Immunol. Today.

[3] Fabbri G, Dalla-Favera R (2016). The molecular pathogenesis of chronic lymphocytic leukaemia. Nat. Rev. Cancer.

[4] Deaglio S (2007). CD38 and ZAP-70 are functionally linked and mark CLL cells with high migratory potential. Blood.

[5] Kriston, C. et al. Low CD23 expression correlates with high CD38 expression and the presence of trisomy 12 in CLL. *Hematol. Oncol*. 35(1):58-63 (2015). 10.1002/hon.2244 26119874

[6] Malavasi F (2011). CD38 and chronic lymphocytic leukemia: a decade later. Blood.

[7] Rombout A, Verhasselt B, Philippe J (2016). Lipoprotein lipase in chronic lymphocytic leukemia: function and prognostic implications. Eur. J. Haematol..

[8] Liu W (2001). Direct inhibition of Bruton’s tyrosine kinase by IBtk, a Btk-binding protein. Nat. Immunol..

[9] Spatuzza C (2008). Physical and functional characterisation of the genetic locus of IBtk, an inhibitor of Bruton’s tyrosine kinase: evidence for three protein isoforms of IBtk. Nucleic Acids Res..

[10] Stilgenbauer S (1999). Incidence and clinical significance of 6q deletions in B cell chronic lymphocytic leukemia. Leukemia.

[11] Dalsass A (2013). 6q deletion detected by fluorescence in situ hybridization using bacterial artificial chromosome in chronic lymphocytic leukemia. Eur. J. Haematol..

[12] Wang DM (2011). Intermediate prognosis of 6q deletion in chronic lymphocytic leukemia. Leuk. Lymphoma.

[13] Broseus J. et al. Relapsed diffuse large B-cell lymphoma present different genomic profiles between early and late relapses. *Oncotarget* 20;7(51):83987-84002 (2016). 10.18632/oncotarget.9793 PMC5356640 27276707

[14] Fiume G (2009). Computational analysis and in vivo validation of a microRNA encoded by the IBTK gene, a regulator of B-lymphocytes differentiation and survival. Comput. Biol. Chem..

[15] Janda E (2011). Btk regulation in human and mouse B cells via protein kinase C phosphorylation of IBtkgamma. Blood.

[16] Pisano A (2015). CRL3IBTK regulates the tumor suppressor Pdcd4 through ubiquitylation coupled to proteasomal degradation. J. Biol. Chem..

[17] Fiume, G. et al. IBTK differently modulates gene expression and RNA splicing in HeLa and K562 cells. *Int. J. Mol. Sci*. **17**(11), 1848 (2016). 10.3390/ijms17111848 PMC5133848 27827994

[18] Luo J (2009). A genome-wide RNAi screen identifies multiple synthetic lethal interactions with the Ras oncogene. Cell.

[19] Kim TH, Shin SW, Park JS, Park CS (2015). Genome wide identification and expression profile in epithelial cells exposed to TiO(2) particles. Environ. Toxicol..

[20] Baird TD (2014). Selective mRNA translation during eIF2 phosphorylation induces expression of IBTKalpha. Mol. Biol. Cell..

[21] Cahill N (2013). 450K-array analysis of chronic lymphocytic leukemia cells reveals global DNA methylation to be relatively stable over time and similar in resting and proliferative compartments. Leukemia.

[22] Mimmi, S. et al. Evidence of shared epitopic reactivity among independent B-cell clones in chronic lymphocytic leukemia patients. *Leukemia* 30(12):2419-2422 (2016). 10.1038/leu.2016.245 PMC5155031 27568521

[23] Ricci F, Tedeschi A, Morra E, Montillo M (2009). Fludarabine in the treatment of chronic lymphocytic leukemia: a review. Ther. Clin. Risk Manag..

[24] Fernandez-Calotti PX, Lopez-Guerra M, Colomer D, Pastor-Anglada M (2012). Enhancement of fludarabine sensitivity by all-trans-retinoic acid in chronic lymphocytic leukemia cells. Haematologica.

[25] Stacchini A (1999). MEC1 and MEC2: two new cell lines derived from B-chronic lymphocytic leukaemia in prolymphocytoid transformation. Leuk. Res..

[26] Henrich S (2011). Fludarabine nucleoside modulates nuclear “survival and death” proteins in resistant chronic lymphocytic leukemia cells. Nucleosides. Nucleotides. Nucleic Acids.

[27] Park HH (2007). The death domain superfamily in intracellular signaling of apoptosis and inflammation. Annu. Rev. Immunol..

[28] Edelmann B (2011). Caspase-8 and caspase-7 sequentially mediate proteolytic activation of acid sphingomyelinase in TNF-R1 receptosomes. EMBO J..

[29] McIlwain DR, Berger T, Mak TW (2013). Caspase functions in cell death and disease. Cold Spring Harb. Perspect. Biol..

[30] Chourasia AH, Macleod KF (2015). Tumor suppressor functions of BNIP3 and mitophagy. Autophagy.

[31] Alhourani E (2016). BIRC3 alterations in chronic and B-cell acute lymphocytic leukemia patients. Oncol. Lett..

[32] Rossi D (2012). Disruption of BIRC3 associates with fludarabine chemorefractoriness in TP53 wild-type chronic lymphocytic leukemia. Blood.

[33] Binet JL (1981). A new prognostic classification of chronic lymphocytic leukemia derived from a multivariate survival analysis. Cancer.

[34] Giordano V (1997). Shc mediates IL-6 signaling by interacting with gp130 and Jak2 kinase. J. Immunol..

[35] Scala G (1994). The expression of the interleukin 6 gene is induced by the human immunodeficiency virus 1 TAT protein. J. Exp. Med..

[36] Albano F (2013). Markers of mitochondrial dysfunction during the diclofenac-induced apoptosis in melanoma cell lines. Biochimie.

[37] Gelzo M (2014). Evaluation of cytotoxic effects of 7-dehydrocholesterol on melanoma cells. Free Radic. Biol. Med..

[38] Fiume, G. et al. Impairment of T cell development and acute inflammatory response in HIV-1 Tat transgenic mice. *Scientific Reports* **5**, 13864 (2015) 10.1038/srep13864 PMC4561375 26343909

