# Supplementary material for: The expression of inhibitor of bruton’s tyrosine kinase gene is progressively up regulated in the clinical course of chronic lymphocytic leukaemia conferring resistance to apoptosis
Source: Cell Death Dis. 2018 Jan 9;9(1):13. doi: 10.1038/s41419-017-0026-3 (PMC5849039; doi:10.1038/s41419-017-0026-3)
Supplement: Supplementary file 1 — Supplementary Informations [file 41419_2017_26_MOESM1_ESM.docx]

**Supplementary Informations**

**Supplementary Table 1. Clinical data and Immunophenotype of CLL patients.**

The list of CLL patients includes: age, sex, Binet-stage, white blood cell (WBC) count at the moment of the sampling, immunophenotype, κ:λ quantification, group assignment, and type of first line therapy (only for In-therapy patients). IT = In-Therapy; R = rituximab; F = fludarabine; C = clorambucil; B = bendamustine; I = Ibrutinib; n.a = not available.

**Supplementary Table 2. Primers of genes analysed by RT-qPCR.**

Forward (FW) and reverse (RV) primers used for analyzing the gene expression of *IBTK*α, *CD38*, *LPL*, *ZAP70*, *TNF*α and β-*ACTIN* by RT-qPCR.

**Supplementary Table 3. Expression analysis of apoptotic genes with or without *IBTK*α RNA interference**

DeFew cells were transduced with shCTRL or sh*IBTK*α, and after 5 days the total RNA was analysed by RT-qPCR, using RT^2^ Profiler PCR Array-Human Apoptosis (Qiagen). Significant difference in gene expression was considered for at least 2-folds up regulation or down-regulation. We considered as “not detected” genes with Ct values ranging from 30 to 40 in shCTRL cells. Data are the mean of 3 independent experiments for each shRNA transduction.

**Supplementary Figure 1. Flow cytometry of isolated CLL cells.**

Flow cytometry of CLL cells (CD19+, CD20+, CD23+, CD5+) isolated from peripheral blood according to the protocol described in Materials and Methods. Purity grade of CLL population of a representative patient (CLL21) is shown.

**Supplementary Figure 2**. **Expression levels of *IBTK*α transcripts in CLL patients.**

Total RNA from healthy donors (HD) and CLL patients of Binet A (CLL9, 13, 16, 21, 29, 33), Binet B (CLL3, 6, 20, 37) and Binet C (CLL30, 46) were analysed by qRT-PCR. Protein extracts of the same CLL samples were analysed by Western blotting in Figure 1E.

**Supplementary Figure 3. Flow cytometry–based detection of IBtkα protein.**

The IBtkα protein was detected by flow cytometry following the staining of CLL cells with anti-IBtkα antibody (ThermoFisher Scientific #PA5-24224). Healthy donor and representative patients of Binet A (CLL13), Binet B (CLL20) and Binet C (CLL46) stages are shown.

***Supplementary Figure 4. IBTK*α silencing by RNA interference.**

DeFew cells were transduced with shCTRL or sh*IBTKα.* (A) Total RNA was analysed by RT-qPCR using *IBTK*α and β-*ACTIN* primers. Values of *IBTK*α expression were normalized to β-*ACTIN*. (B) Protein extracts were separated by 4-12% NuPAGE Novex Gels (ThermoFisher Scientific) and analysed by Western blotting with antibodies against IBtkα (ThermoFisher Scientific #PA5-24224) and β-actin (Cell Signaling #3700S). *n.s.* indicates non-specific bands.
