# Supplementary material for: The expression of inhibitor of bruton’s tyrosine kinase gene is progressively up regulated in the clinical course of chronic lymphocytic leukaemia conferring resistance to apoptosis
Source: Cell Death Dis. 2018 Jan 9;9(1):13. doi: 10.1038/s41419-017-0026-3 (PMC5849039; doi:10.1038/s41419-017-0026-3)
Supplement: Supplementary file 3 — Supplementary Table 2 [file 41419_2017_26_MOESM3_ESM.docx]

|  |  |
| --- | --- |
| **Primer** | **Sequence (5’ - 3’)** |
| *IBTKα - FW* | CCTCCTGTTGTGGATCTCAGAACTAT |
| *IBTKα - RV* | GAGAAAGTTTAACTCCATGAGAAAC |
| *β-ACTIN - FW* | CCGACAGGATGCAGAAGGAGA |
| *β-ACTIN - RV* | CGTCATACTCCTGCTTGCTG |
| *CD38 - FW* | GCTCAATGGATCCCGCAGTA |
| *CD38 - RV* | GGATCCTGGCATAAGTCTCTGG |
| *LPL - FW* | GCTGGACGGTAACAGGTAACAGGAATG |
| *LPL - RV* | AGCCAGTCCACCACAATGAC |
| *ZAP70 - FW* | GTACCACAGCAGCCTGACG |
| *ZAP70 - RV* | ATGTGCCCTGCTCCTTCC |
| *TNFα - FW* | AGCCCATGTTGTAGCAAACC |
| *TNFα - RV* | TGAGGTACAGGCCCTCTGAT |
|  |  |
