# Supplementary material for: The expression of inhibitor of bruton’s tyrosine kinase gene is progressively up regulated in the clinical course of chronic lymphocytic leukaemia conferring resistance to apoptosis
Source: Cell Death Dis. 2018 Jan 9;9(1):13. doi: 10.1038/s41419-017-0026-3 (PMC5849039; doi:10.1038/s41419-017-0026-3)
Supplement: Supplementary file 4 — Supplementary Table 3 [file 41419_2017_26_MOESM4_ESM.docx]

| **Gene Symbol** | **DeFew shCTRL mRNA expression (Mean value ± SE)** | | | **DeFew sh*IBTK*α mRNA expression (Mean value ± SE)** | | | **DeFew sh*IBTK*α versus shCTRL Up/Down-regulated** |
| --- | --- | --- | --- | --- | --- | --- | --- |
| ABL1 | 0,00461 | ± | 0,00078 | 0,00543 | ± | 0,00092 | - |
| AIFM1 | 0,02066 | ± | 0,00351 | 0,02549 | ± | 0,00433 | - |
| AKT1 | 0,00354 | ± | 0,00060 | 0,00562 | ± | 0,00096 | - |
| APAF1 | 0,00248 | ± | 0,00042 | 0,00421 | ± | 0,00072 | - |
| BAD | 0,00060 | ± | 0,00010 | 0,00065 | ± | 0,00011 | - |
| BAG1 | 0,00175 | ± | 0,00030 | 0,00156 | ± | 0,00027 | - |
| BAG3 | 0,00604 | ± | 0,00103 | 0,00947 | ± | 0,00161 | - |
| BAK1 | 0,00489 | ± | 0,00083 | 0,00677 | ± | 0,00115 | - |
| BAX | 0,00201 | ± | 0,00034 | 0,00213 | ± | 0,00036 | - |
| BCL10 | 0,00628 | ± | 0,00107 | 0,00645 | ± | 0,00110 | - |
| BCL2 | 0,00244 | ± | 0,00041 | 0,00185 | ± | 0,00031 | - |
| BCL2A1 | 0,03621 | ± | 0,00616 | 0,04159 | ± | 0,00707 | - |
| BCL2L1 | 0,00542 | ± | 0,00092 | 0,00526 | ± | 0,00089 | - |
| BCL2L10 | Not detected | | | Not detected | | | - |
| BCL2L11 | 0,00253 | ± | 0,00043 | 0,00295 | ± | 0,00050 | - |
| BCL2L2 | 0,00669 | ± | 0,00114 | 0,00806 | ± | 0,00137 | - |
| BFAR | 0,01157 | ± | 0,00197 | 0,01672 | ± | 0,00284 | - |
| BID | 0,00144 | ± | 0,00024 | 0,00183 | ± | 0,00031 | - |
| BIK | 0,00029 | ± | 0,00005 | 0,00036 | ± | 0,00006 | - |
| BIRC2 | 0,02342 | ± | 0,00398 | 0,03425 | ± | 0,00582 | - |
| BIRC3 | 0,05214 | ± | 0,00886 | 0,11129 | ± | 0,01892 | Up-regulated |
| BIRC5 | 0,00046 | ± | 0,00008 | 0,00058 | ± | 0,00010 | - |
| BIRC6 | 0,01168 | ± | 0,00199 | 0,01362 | ± | 0,00232 | - |
| BNIP2 | 0,01054 | ± | 0,00179 | 0,01407 | ± | 0,00239 | - |
| BNIP3 | 0,00977 | ± | 0,00166 | 0,02389 | ± | 0,00406 | Up-regulated |
| BNIP3L | 0,01480 | ± | 0,00252 | 0,02177 | ± | 0,00370 | - |
| BRAF | 0,00453 | ± | 0,00077 | 0,00544 | ± | 0,00092 | - |
| CASP1 | 0,06838 | ± | 0,01162 | 0,07186 | ± | 0,01222 | - |
| CASP10 | 0,00137 | ± | 0,00023 | 0,00118 | ± | 0,00020 | - |
| CASP14 | Not detected | | | Not detected | | | - |
| CASP2 | 0,01165 | ± | 0,00198 | 0,01992 | ± | 0,00339 | - |
| CASP3 | 0,01155 | ± | 0,00196 | 0,01620 | ± | 0,00275 | - |
| CASP4 | 0,01591 | ± | 0,00270 | 0,02348 | ± | 0,00399 | - |
| CASP5 | 0,00232 | ± | 0,00040 | 0,00325 | ± | 0,00055 | - |
| CASP6 | 0,00355 | ± | 0,00060 | 0,00576 | ± | 0,00098 | - |
| CASP7 | 0,00485 | ± | 0,00082 | 0,01063 | ± | 0,00181 | Up-regulated |
| CASP8 | 0,00669 | ± | 0,00114 | 0,00756 | ± | 0,00129 | - |
| CASP9 | 0,01408 | ± | 0,00239 | 0,01805 | ± | 0,00307 | - |
| CD27 | 0,00243 | ± | 0,00041 | 0,00272 | ± | 0,00046 | - |
| CD40 | 0,00345 | ± | 0,00059 | 0,00294 | ± | 0,00050 | - |
| CD40LG | 0,00043 | ± | 0,00007 | 0,00035 | ± | 0,00006 | - |
| CD70 | 0,01073 | ± | 0,00182 | 0,00910 | ± | 0,00155 | - |
| CFLAR | 0,00542 | ± | 0,00092 | 0,00437 | ± | 0,00074 | - |
| CIDEA | Not detected | | | Not detected | | | - |
| CIDEB | 0,00911 | ± | 0,00155 | 0,00870 | ± | 0,00148 | - |
| CRADD | 0,00165 | ± | 0,00028 | 0,00376 | ± | 0,00064 | Up-regulated |
| CYCS | 0,00913 | ± | 0,00155 | 0,00931 | ± | 0,00158 | - |
| DAPK1 | Not detected | | | Not detected | | | - |
| DFFA | 0,01273 | ± | 0,00216 | 0,01690 | ± | 0,00287 | - |
| BIABLO | 0,00068 | ± | 0,00011 | 0,00065 | ± | 0,00011 | - |
| FADD | 0,00193 | ± | 0,00033 | 0,00194 | ± | 0,00033 | - |
| FAS | 0,01049 | ± | 0,00178 | 0,01433 | ± | 0,00244 | - |
| FASLG | 0,00021 | ± | 0,00003 | 0,00016 | ± | 0,00001 | - |
| GADD45A | Not detected | | | Not detected | | | - |
| HRK | 0,00346 | ± | 0,00059 | 0,00425 | ± | 0,00072 | - |
| IGF1R | 0,01246 | ± | 0,00212 | 0,01436 | ± | 0,00244 | - |
| IL10 | 0,00068 | ± | 0,00012 | 0,00046 | ± | 0,00008 | - |
| LTA | 0,00363 | ± | 0,00062 | 0,00509 | ± | 0,00087 | - |
| LTBR | 0,00021 | ± | 0,00004 | 0,00022 | ± | 0,00004 | - |
| MCL1 | 0,07218 | ± | 0,01227 | 0,11059 | ± | 0,01880 | - |
| NAIP | 0,01240 | ± | 0,00211 | 0,01452 | ± | 0,00247 | - |
| NFKB1 | 0,00610 | ± | 0,00104 | 0,00619 | ± | 0,00105 | - |
| NOD1 | 0,00102 | ± | 0,00017 | 0,00174 | ± | 0,00030 | - |
| NOL3 | Not detected | | | Not detected | | | - |
| PYCARD | 0,00084 | ± | 0,00014 | 0,00109 | ± | 0,00019 | - |
| RIPK2 | 0,00703 | ± | 0,00120 | 0,00824 | ± | 0,00140 | - |
| TNF | 0,00157 | ± | 0,00027 | 0,00311 | ± | 0,00053 | Up-regulated |
| TNFRSF10A | Not detected | | | Not detected | | | - |
| TNFRSF10B | 0,00676 | ± | 0,00115 | 0,00708 | ± | 0,00120 | - |
| TNFRSF11B | Not detected | | | Not detected | | | - |
| TNFRSF1A | Not detected | | | Not detected | | | - |
| TNFRSF1B | Not detected | | | Not detected | | | - |
| TNFRSF21 | 0,00532 | ± | 0,00090 | 0,00921 | ± | 0,00157 | - |
| TNFRSF25 | Not detected | | | Not detected | | | - |
| TNFRSF9 | 0,00274 | ± | 0,00047 | 0,00107 | ± | 0,00018 | - |
| TNFSF10 | 0,00759 | ± | 0,00129 | 0,01256 | ± | 0,00214 | - |
| TNFSF8 | 0,00043 | ± | 0,00007 | 0,00076 | ± | 0,00013 | - |
| TP53 | 0,01617 | ± | 0,00275 | 0,01985 | ± | 0,00337 | - |
| TP53B2 | 0,01342 | ± | 0,00228 | 0,01716 | ± | 0,00292 | - |
| TP73 | Not detected | | | Not detected | | | - |
| TRADD | Not detected | | | Not detected | | | - |
| TRAF2 | 0,00066 | ± | 0,00011 | 0,00075 | ± | 0,00013 | - |
| TRAF3 | 0,00902 | ± | 0,00153 | 0,01058 | ± | 0,00180 | - |
| XIAP | 0,00587 | ± | 0,00100 | 0,00498 | ± | 0,00085 | - |
