# Supplementary figures and images for: The expression of inhibitor of bruton’s tyrosine kinase gene is progressively up regulated in the clinical course of chronic lymphocytic leukaemia conferring resistance to apoptosis
Source: Cell Death Dis. 2018 Jan 9;9(1):13. doi: 10.1038/s41419-017-0026-3 (PMC5849039; doi:10.1038/s41419-017-0026-3)

## Supplementary Figure 1

**A**

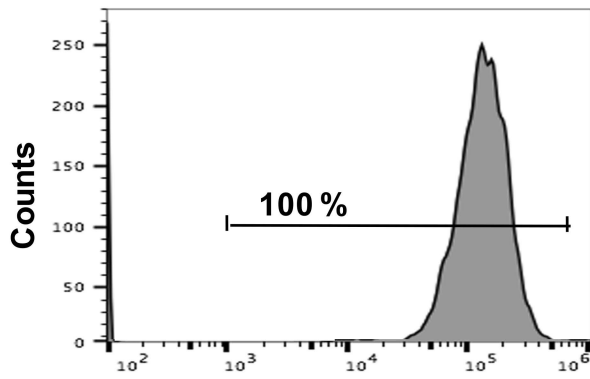

**CD19**

**B**

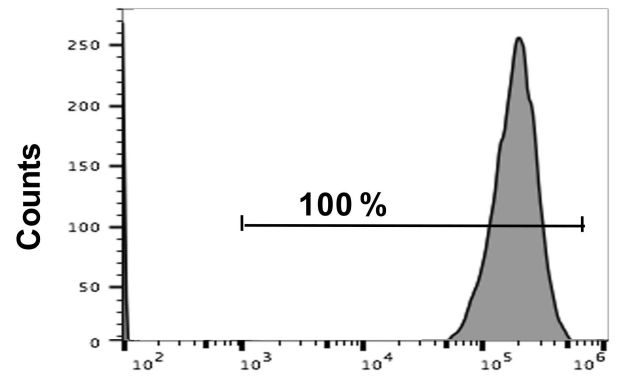

**CD5**

**C**

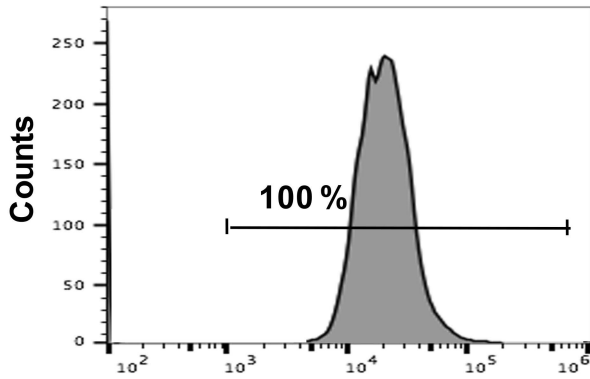

**CD20**

**D**

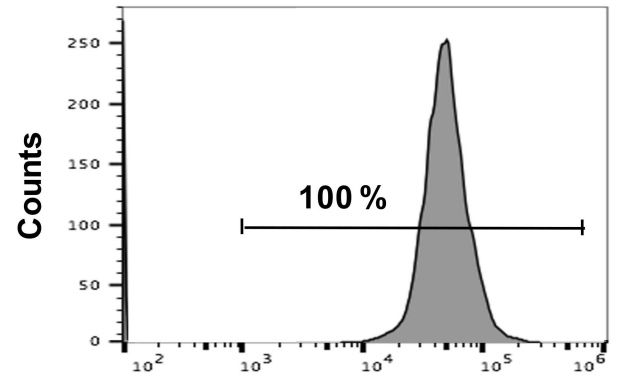

**CD23**

Supplement: Supplementary file 5 — Supplementary Figure 1 [file 41419_2017_26_MOESM5_ESM.pdf]

Supplementary Figure 2

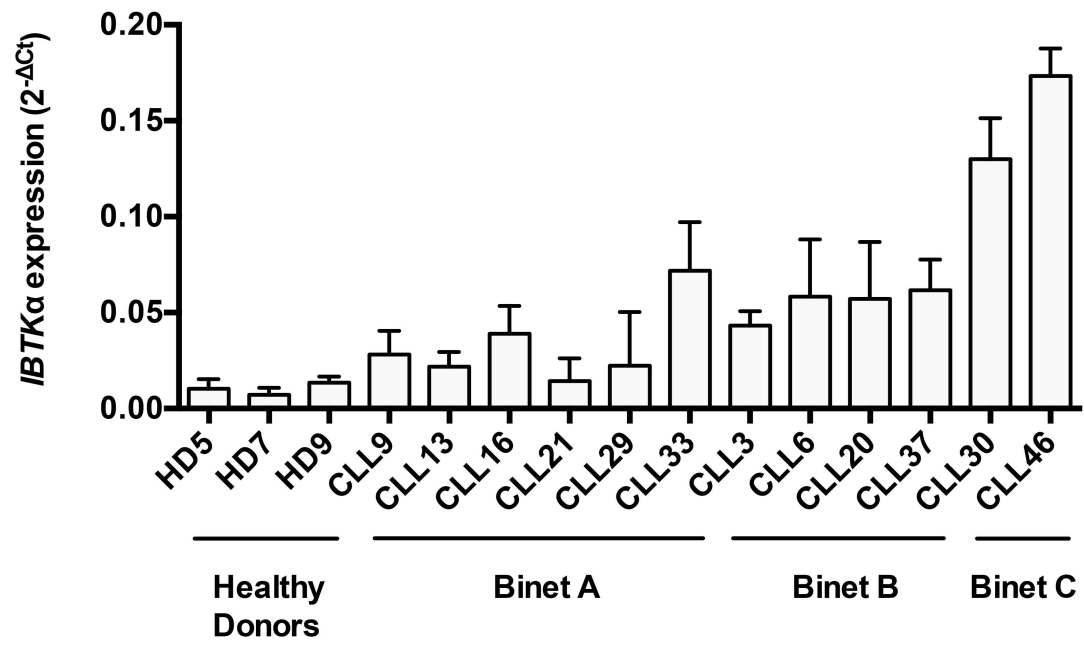

Supplement: Supplementary file 6 — Supplementary Figure 2 [file 41419_2017_26_MOESM6_ESM.pdf]

Supplementary Figure 3

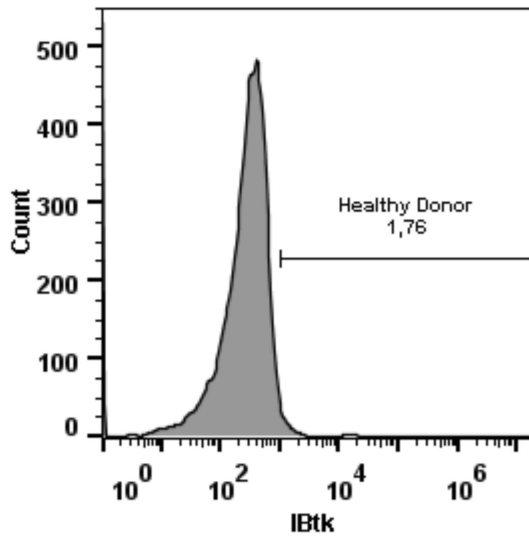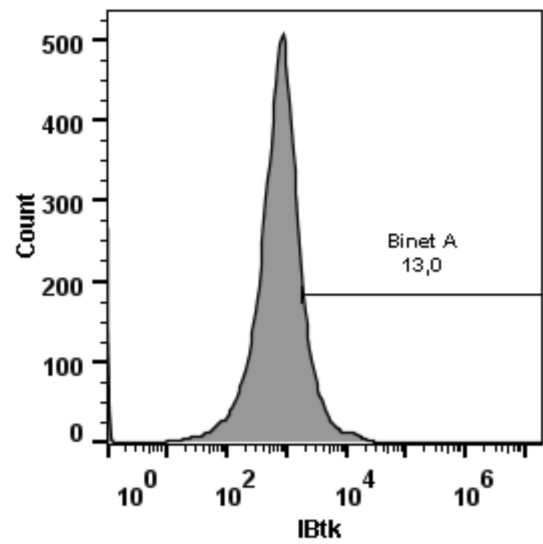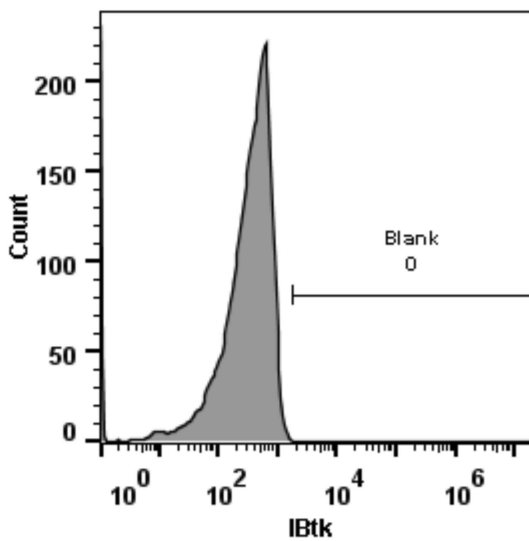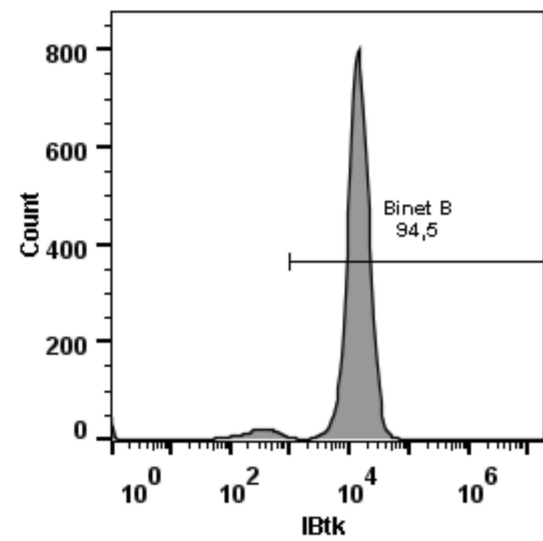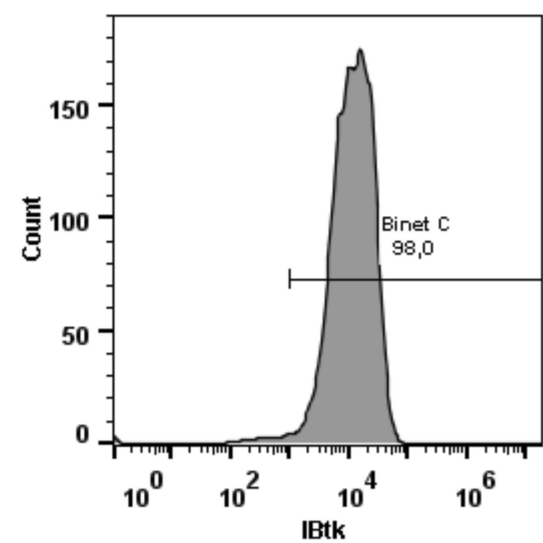

Supplement: Supplementary file 7 — Supplementary Figure 3 [file 41419_2017_26_MOESM7_ESM.pdf]

Supplementary Figure 4

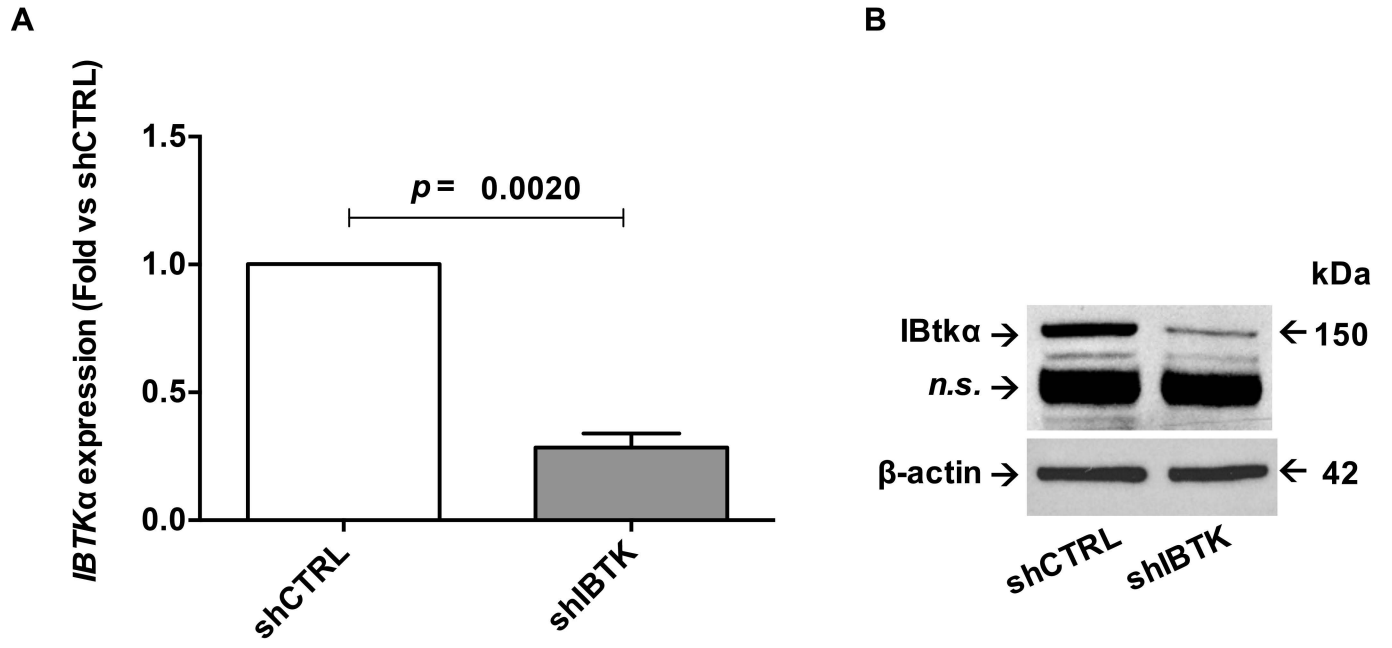

Supplement: Supplementary file 8 — Supplementary Figure 4 [file 41419_2017_26_MOESM8_ESM.pdf]
